# Eliminating the Brittleness Constituent to Enhance Toughness of the High-Strength Steel Weld Heat-Affected Zone Using Electropulsing

**DOI:** 10.3390/ma15062135

**Published:** 2022-03-14

**Authors:** Zhanglan Chen, Yunfeng Xiong, Xiaowen Li, Zongmin Li

**Affiliations:** School of Marine Engineering, Jimei University, Xiamen 361021, China; zhanglanchen@jmu.edu.cn (Z.C.); 2006610000057@jmu.edu.cn (X.L.); 200661000057@jmu.edu.cn (Z.L.)

**Keywords:** heat-affected zone, martensite–austenite constituent, evolution, electropulsing treatment, toughness enhancement

## Abstract

The evolution of the martensite–austenite (MA) constituent in the heat-affected zone (HAZ) of high-strength steel FH690 welds when subjected to electropulsing (EP) treatment was investigated herein, with the aim of eliminating brittle MA to enhance toughness. The features induced by EPT were correlated with the microstructure and fractography through scanning electron microscopy and electron backscatter diffraction analyses, together constituting an impact property evaluation. The Charpy V-notch impact results showed EPT could improve toughness of the HAZ from 34.1 J to 51.8 J (the calibrated value was 46 J). Examinations of EP-treated microstructure showed a preferred Joule heating: at the site of the MA constituent, the cleavage fractography introduced by the MA constituent was substituted with ductile dimples with various sizes. Decreases in grain size of 40% and 47% for the matrix and the retained austenite, respectively, were achieved; while for regions without the MA constituent, microstructural modification was negligible. The temperature rise at sample surface was less than 60 °C. The mechanism behind this favorable Joule heating for the MA constituent was correlated with the electrical properties of the MA constituent in contrast with martensite matrix. The toughness enhancement of the HAZ was thus attributed to the elimination of the coarse MA constituent. The present investigation suggested that electropulsing, characterized as a narrow-duration current, is a promising method for preferred elimination of brittle factors and thus improving the toughness of HAZ of high-strength steel within a limited region with a width less than 2 mm.

## 1. Introduction

High-strength steel (HSS) is a promising material for lightweight applications, especially in oceanic engineering, in which the mechanical properties of high strength and good toughness are required. Welding is an effective connection technique, but generates steep thermal gradients in the heat-affected zone (HAZ). In addition to commonly observed cold cracking and softening, the HAZ of HSSs also suffers from undesirable features due to the critical cooling speed resulting from high alloyed elements. A martensite–austenite (MA) constituent is a typical heterogeneous feature arising from postwelding fast cooling [1].

The mechanical behavior of an MA constituent is different from that of the matrix, which creates a local inhomogeneous strain, promoting the nucleation of microvoids at the MA interfaces and degrading the HAZ’s fracture toughness. According to the classical Griffith theory [2,3], a brittle fracture occurs once the area of MA constituents reaches a critical value, such as 1 μm^2^ [4]. This local brittle fracture remains ineffectively suppressed by narrowing the HAZ region with decreased heat input in in situ welds [1]. Offline treatment is used as an alternative in order to improve the toughness of the HAZ.

Several offline processes have previously been proposed to suppress the MA constituent, such as postwelding heat treatment and alloying. Xie et al. [5] introduced a tempering process at low temperature and found that the toughness was significantly improved due to the decomposition of the MA constituent. Intercritical normalizing between normalizing and tempering was investigated to refine MA constituents, and a significant toughness enhancement was achieved [6,7]. Luo et al. [8] applied ausforming to enable the microstructure modification of an MA constituent, and concluded the ausforming contributed to the austenite formation within the MA constituent, which was helpful in improving the toughness. Since HAZ is limited to a very narrow region, general heat treatments readily generate a region beyond the original weld-HAZ, which may pollute the microstructure adjacent to the HAZ. Li et al. [9] achieved superior toughness of the HAZ in steels by adding 0.006 wt % Nb, which introduced high-angle grain boundaries. However, the use of an extra alloying element is not always cost-effective. A toughness of the HAZ compatible with the HSS base metal remains a challenge for a confident weld joint.

Electropulsing (EP) has been widely applied to enhance the mechanical properties of steels. With the advantages of rapid heating induced by high-intensity EP, as compared to a general heat source, a large degree of overheating and undercooling can be obtained, which results in a high nucleation rate of austenite, and therefore a refined microstructure. EP showed a good ability to quench and temper 40Cr steel [10] and 42CrMo steel [11], and to remove residual stress in welds [12]. EP was also applied to crack healing by melting the crack location [13]. However, the application of EP with the aim to enhance toughness of HSS without polluting the neighboring microstructure, to the best of our knowledge, has seldom been reported. It remains unknown whether the evolution of an MA constituent under EP is beneficial for toughness enhancement.

The electrical resistivity of an MA constituent is higher than that of the HAZ matrix [14]. When exposed to an electrical current, an MA constituent produce a higher Joule heat. This can be advantageously exploited to differentiate the instantaneous thermal cycle if the EP parameters are properly selected. Herein, the response of the MA and the HAZ matrix of F690 steel to EP treatment (EPT) was analyzed through mechanical property tests, fractography, and microstructural examinations. Such research has not been conducted previously; hence, this study provides new insight into the application of EP and HAZ toughness enhancement.

## 2. Materials and Methods

**Figure 1:**
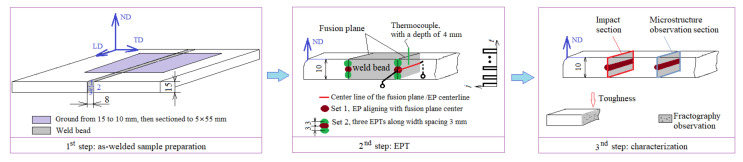
Schematic of the EPT process flowchart.

To investigate EP’s effect on the MA constituent, HAZ samples containing MA constituents were fabricated and sectioned. Then, weld fusion lines were revealed for EPT and microstructure observation. After EPT, the fracture surfaces were correlated with the toughness and microstructure, as schematically shown in Figure 1.

### 2.1. Material and Weld Procedure

FH690 ship steel subjected to a thermomechanical control process (TMCP) was used as the test material in this study. FH690 is a typical low-carbon steel, and its chemical composition, determined by optical emission spectroscopy, is presented in Table 1. The carbon equivalent was calculated to be 0.35% according to the following equation: $C_{eq}=C+\mathrm{Mn}/6+\mathrm{Si}/24$ [15]. The yield strength and toughness of FH 690 were measured as 690 MPa and 0.46 J/mm^2^, respectively. According to the formula in [16], the starting temperature (*A_C_*_1_) and completion temperature (*A_C_*_3_) for austenitization of FH690 were estimated to be 699 °C and 847 °C, respectively.

The experimental schedule is shown in Figure 1. Plates with a 15 mm thickness were joined by hybrid gas (20% Ar and 80% CO_2_)-shielded welding with a Megafil 690R E110-111T1 (AWS code, φ1.2 mm) weld filler (Stein, Berlin, German). Since intercritical reheating is more susceptible to the formation of an MA constituent in multipass welding [1,9], a square groove with a three-pass configuration was designed to generate a HAZ plane parallel to the impact load direction compatible with EP operation, as shown in the first panel of Figure 1. This groove also provided comparable samples for both impact toughness and microstructure investigations. The dimension of the butt-welded specimens was 350 mm × 100 mm. The weld parameters for each pass were as follows: current 110–120 A, arc voltage 20–22 V, heat input 2.58 kJ/mm, and gas flow rate 22 L/min. The temperature between each weld pass was controlled to 100–150 °C at the middle of weld bead using a noncontact infrared thermometer.

After welding, the welded workpieces were ground to 10 mm by removing the last weld bead. Then, 20 sub-size impact testing samples with thickness × width × length of 5 mm × 10 mm × 55 mm were extracted by using electrical-discharge machining (EDM, Rixin spark machine, Shenzhen, China).

### 2.2. EP Treatment

**Figure 2:**
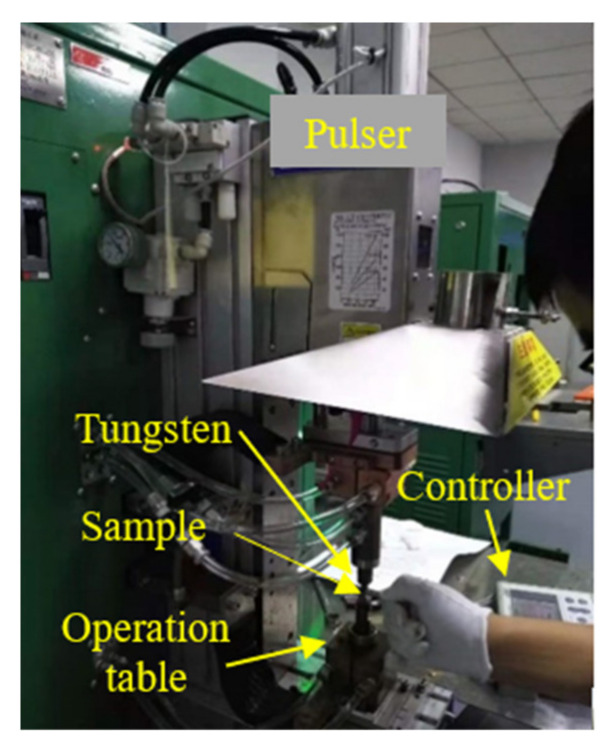
EPT equipment.

Before EP treatment (EPT), a 2% nital solution was used to etch the HAZ to reveal the fusion line. The EPT parameters included current density, pulse duration, and frequency, which were determined by the consideration of toughness improvement under electric breakdown. After repeated trials in our preliminary investigations, the EP parameters were optimized to: current density *j* = 33 × 10^6^ A/m^2^, pulse duration *t_d_* = 30 × 10^−3^ s, and a frequency of 0.9 Hz. EPT was performed using a MIYACHI is-800A current pulser (Amada, Shanghai, China), as shown in Figure 2. The EP parameters were programmable by the controller. The current was conducted through the sample thickness of 5 mm by a tungsten electrode with a diameter of 5 mm. The current density was monitored during EPT with an oscilloscope.

Since EPT could not cover the sample width due to the electrode diameter being shorter than the sample width (10 mm), two sets of samples were prepared (each set contained 10 samples): Set 1 underwent EPT once with the electrode centerline (EP-center) aligned with that of the fusion plane; and Set 2 underwent three consecutive EPTs spaced at 3 mm to fully cover the sample width, as shown in the second panel in Figure 1.

EPT introduced a temperature rise, measured by a K-type thermocouple at the point offsetting electrode center by 1 mm, which read about 321 °C, referring to a rough understanding of the EP-introduced Joule heating. The temperature rise at sample surface, measured by an infrared thermometer, was 53 °C.

### 2.3. Microstructural Characterization and Mechanical Properties

V-shaped notches were machined transversely, aligning the fusion plane of each sample according to the rules of the China classification society [15]. Since the samples were subsized, a pad was placed onto the impact tester to elevate samples to the same height as the standard sample. Charpy impact tests were then performed at –60 °C. The impact toughness values were collected. For comparison, the impact toughness of the as-welded HAZ was also collected. Microhardness measurements were performed with a load of 1000 g and a 5 s dwell time. The fracture morphology was examined by scanning electron microscopy (SEM, 3700, Zeiss, Berlin, Germany) at 5.0 kV.

Microstructure specimens were cut from the HAZ on the side opposite to that used for the impact test. Electron backscatter diffraction (EBSD) specimens were mounted, ground, and electrolytically polished according to the standard metallography procedure. Microscopy observation and phase identification were performed at an accelerating voltage of 20 kV, with step sizes of 0.6 μm and 0.06 μm, respectively.

## 3. Results

### 3.1. Microstructural Examination

**Figure 3:**
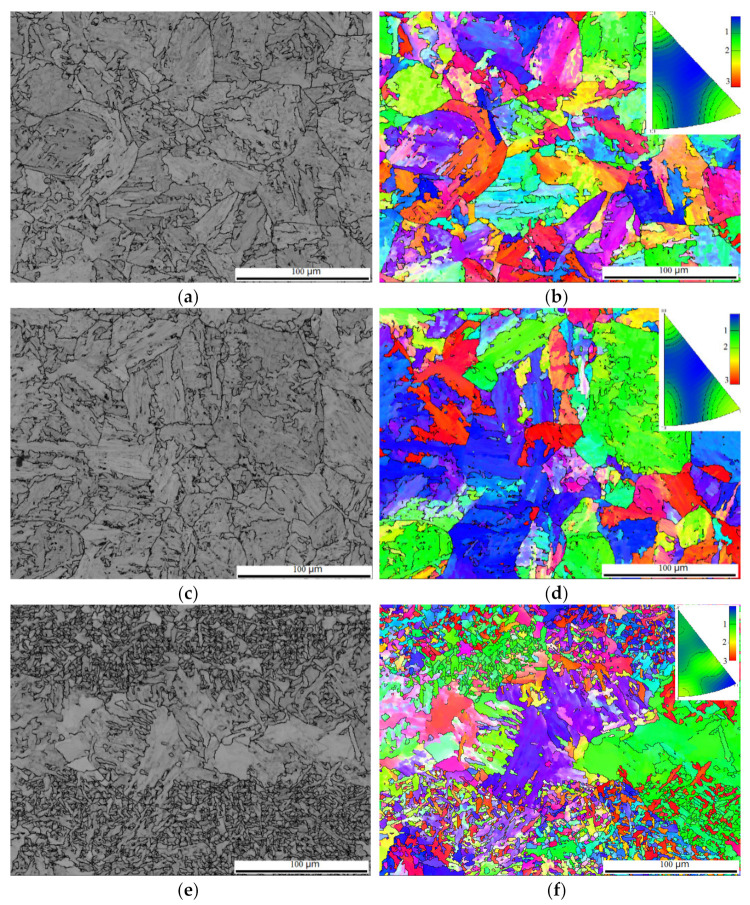
Image quality and orientation image of (**a**,**b**) the as-welded; (**c**,**d**) the typical; and (**e**,**f**) the spotty cases, respectively.

Figure 3 shows the EBSD image quality and corresponding orientation image maps. The microstructure of the as-welded HAZ was predominantly martensite, as shown in Figure 3a. Along the EP centerline, two regions of microstructure were detected, labelled typical and spotty, corresponding to relatively homogeneous martensite similar to the as-welded HAZ, as shown in Figure 3c, and coarse grains surrounded with agglomerated particles, as shown in Figure 3e. The coarse grains turned out to be a thin film parallel to the sample surface. The film was so thin that a metallurgy preparation could remove it. No intensive misorientation was observed, as shown in Figure 3b,d,f.

### 3.2. Phase Distribution

**Figure 4:**
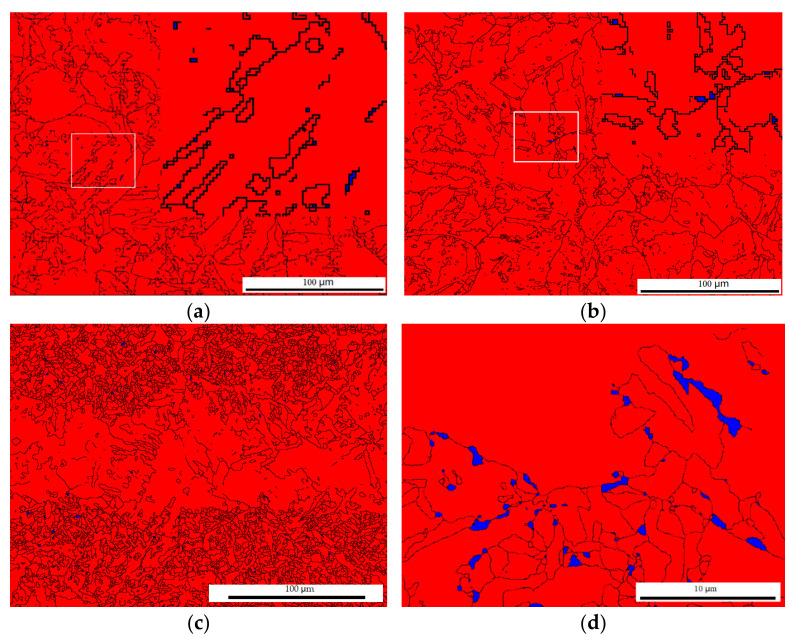
Phase image of the (**a**) as-welded; (**b**) typical; and (**c**) spotty cases and (**d**) magnification of (**c**).

Phase analysis corresponding to the microstructure shown in Figure 3 was carried out by using EBSD. In the as-welded case shown in Figure 4a, face-centered cubic (FCC, γ—Fe or austenite) was observed to be in a scattered and isolated distribution with a fraction less than 0.10%. In the typical EP-treated case, austenite was distributed along grain boundaries with a small fraction of 0.17%, slightly higher than that of the as-welded case, as shown in Figure 4b. Whereas in the spotty case, the austenite fraction is also distributed along grain boundaries with a fraction of 0.45%, obviously higher than that of the as-welded and the typical cases, as shown in Figure 4c. This fraction climbed to 4.62% in a magnified picture, as shown in Figure 4d, indicating a highly concentrated distribution of austenite.

**Figure 5:**
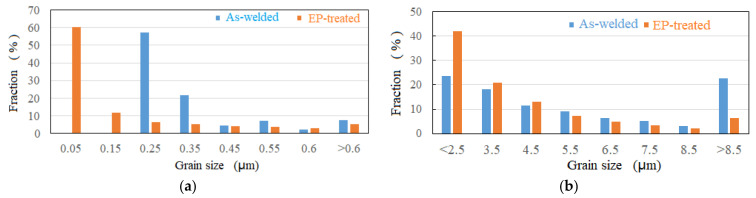
Grain size distributions: (**a**) FCC; (**b**) total.

Since the size of the austenite grain has frequently been reported to have a significant influence on toughness, with a critical size of 0.56 μm [4], the austenite grain size in Figure 4a,d was investigated using EBSD, as shown in Figure 5a. The average size of austenite decreased by 47%, from 0.34 μm to 0.18 μm. Figure 4d shows a 5.32% fraction of FCC with sizes larger than 0.56 μm, whereas in the as-welded case, the counterpart read 7.59%. Figure 5b shows the statistical distribution of the total grain size. It is clear that EPT was helpful to microstructural refinement, with an average grain size of 40%, from 7.07 μm to 4.26 μm; while the microstructural average size shown in Figure 3c was 6.97 μm, a slight decrease compared to the as-welded case. The microstructural refinement shown in Figure 5 suggested the occurrence of recrystallization or austenitization during EPT. In fact, EPT has been reported to generate high heating speed, estimated to be 10,700 °C/s in this study, introducing overheating and a high austenite nucleation rate [10,11], leading to microstructural refinement.

### 3.3. Fractography

**Figure 6:**
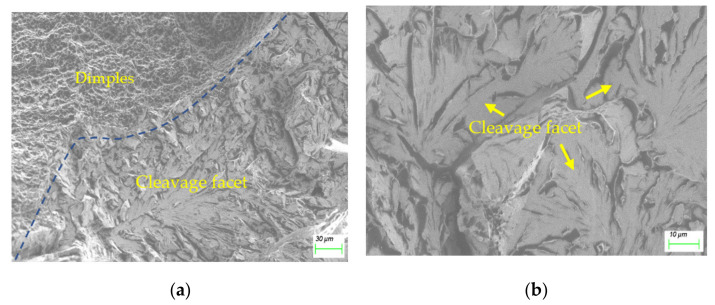
Fractography of: (**a**) fusion plane; (**b**) magnified MA.

For comparison, the typical fractography of the HAZ consisting of MA constituents is displayed in Figure 6a, and locally magnified in Figure 6b. This cleavage morphology is common in MA-introduced brittleness.

**Figure 7:**
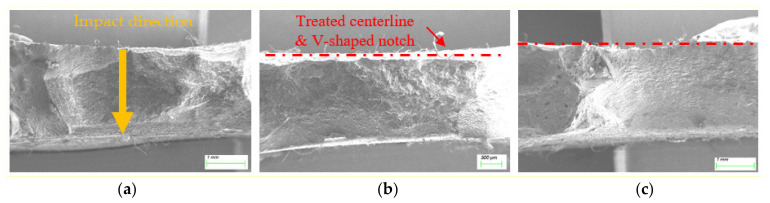
Fracture surfaces (**a**) and (**b**) deviating from the EP-treated centerline; and (**c**) full of poles.

**Figure 8:**
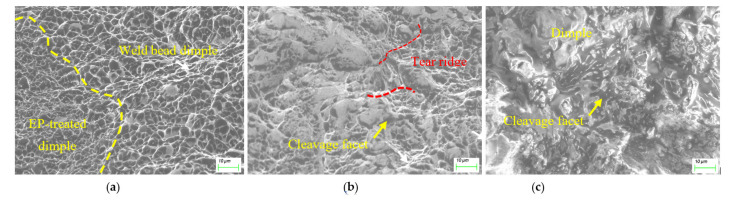
HAZ fracture morphology located at: (**a**) the EP centerline; (**b**) 0.8 mm; (**c**) 1.1 mm away from the EP centerline.

It was difficult to obtain the HAZ MA evolution after EPT, because the fracture surfaces were fairly uneven, completely deviating from the V-shaped notch centerline plane, as displayed in Figure 7a,b. In fact, among 20 EP-treated HAZ samples, only 6 fractured across the notch centerline plane. In addition, five samples were full of pole (features of the weld bead other than the HAZ), with a typical example shown in Figure 7c, and only one sample fracture surface in Set 1 was exactly located at the notch centerline plane (i.e., EP-treated HAZ), as shown in Figure 8.

As shown in Figure 8a, the fractography was full of dimples with scattered sizes, which was different from the weld bead, with its relatively uniform size of dimples, indicating heterogeneous heating along the EPT centerline. The refiner dimple morphology coincided with the finer grain shown in Figure 5b. Figure 8b shows the morphology at a distance from the EPT centerline of about 0.8 mm. The fracture surface appeared to have cleavage facets among the dimples, and the cleavage facets became larger at a further distance away from the EPT centerline of 1.1 mm, as shown in Figure 8c. It was clear that the existence of MA at a certain distance away from EP centerline led to brittle fracture, suggesting that the EPT had an effective region, such as 1.1 mm.

### 3.4. Mechanical Performance

**Figure 9:**
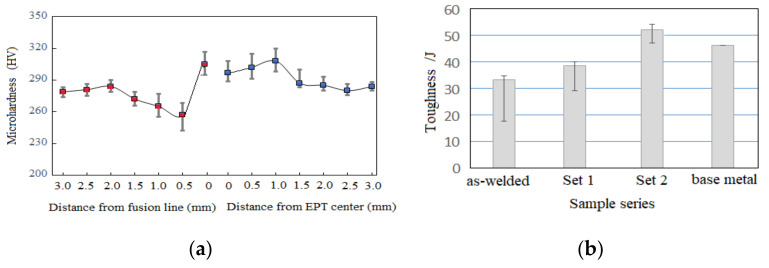
(**a**) Microhardness of Set 1; (**b**) impact toughness.

Figure 9a shows the hardness of the welded joints. The hardness of the EPT centerline was 297 HV_5_, then climbed to the highest value recorded of 308 HV_5_, and the converged to a value similar to the base metal of 285 HV_5_, corresponding to distances from the centerline of 1.0 and 2.0 mm, respectively. As compared to the as-welded counterpart, the HAZ softening was much improved. The refined martensite matrix shown in Figure 4b contributed to the improvement in hardness in terms of Hall–Petch relation [17,18,19]. Meanwhile, Figure 9a shows the occurrence of softening at locations of 0.5–1.5 mm away from fusion line (marked in red), which coincided with the toughness of the distance dependency shown in Figure 8b,c.

Since subsize impact samples were used here, the measured toughness values were normalized by multiplying by a coefficient of 1.5, according to the CCS [14]. Figure 9b displays four sets of toughness data: the as-welded, Set 1, Set 2, and the base metal for comparison. The toughness of the two EP-treated sets was higher than that of the as-welded case, highlighting that EPT was an effective way to improve the toughness of the HAZ. The impact toughness (51.8 J) in the case of Set 2 was higher than that of Set 1, suggesting that toughness enhancement through EPT was effective within a limited width. This gradient result coincided with the distance-dependency hardness (shown in Figure 9a) and dimples (shown in Figure 8). This showed that the deviation of fracture path during impact tests was due to the eccentric MA, as shown in Figure 7.

## 4. Discussion

### 4.1. Mechanism of Toughness Improvement

Figure 9 shows an improved toughness and hardness after EPT. The refined microstructure and improved homogeneous distribution of both the microstructure and FCC phase, shown in Figure 4b and Figure 5, respectively, were contributors in terms of the Hall–Petch equations. On the other hand, the microstructure feature of blocky and island FCC, as shown in Figure 4a, coinciding with the fractography shown in Figure 6, were undetected after the EPT, suggesting this blocky and island MA feature was responsible for the lower toughness of the as-welded HAZ. This result was in agreement with some other studies [20,21] in which thin-film austenite was reported, but was contrary to another study [8] in which blocky austenite surrounded by martensite was found to be more effective in hindering crack propagation.

**Figure 10:**
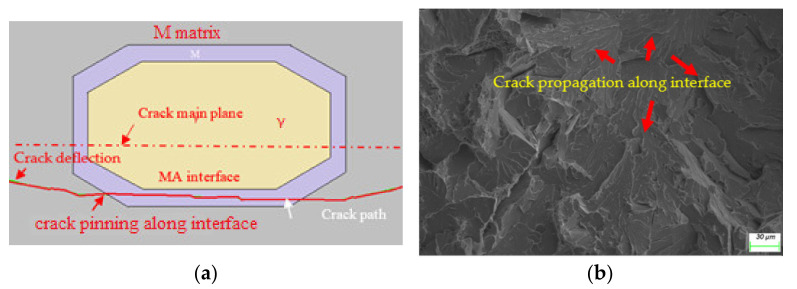
Brittle morphology induced by blocky MA: (**a**) numerical simulation; and (**b**) its experimental counterparts.

To identify the degradation of the blocky MA constituent on toughness, microcracking evolution in terms of fracture mechanics was modelled by combining the elastoplastic finite element and extended finite element methods using the Abaqus package. As shown in Figure 10a, the microstructure with blocky MA constituents; i.e., RA, was surrounded by martensite [1]. Before initiation of the TRIP effect when subjected to plastic deformation, the crack was preferentially deflected by hard martensite and then arrested due to a large deformation of soft austenite, and then propagated along the interface between the hard martensite and the TRIP-introduced martensite, revealing a typical cleavage and intergranular morphology along the prior austenite grain boundary, as shown in Figure 10b and Figure 6. This numerical path was also in agreement with the results of an interrupted three-point bending test in which the crack was captured at the MA interface shown as a solid line in Figure 10a, instead of cracking through the MA/matrix along the crack main plane shown as the red centerline [22].

**Figure 11:**
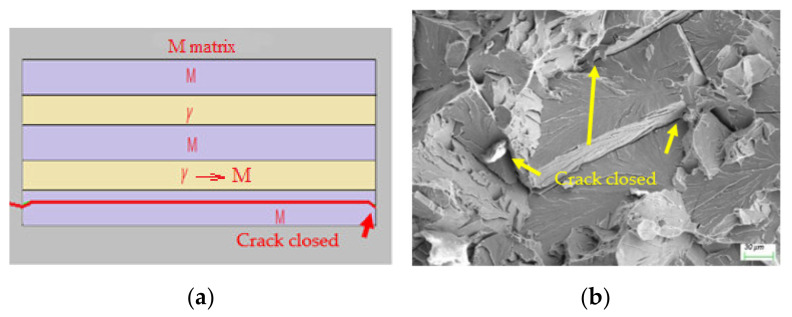
Morphology induced by thin-film MA: (**a**) numerical simulation; and (**b**) the corresponding experimental counterparts.

In contrast, in the case of thin-film or lamellar MA constituents, the crack also preferentially advanced along the MA interface due to soft austenite, then propagated along martensite without further attraction of soft austenite (disabled by the TRIP effect), until it was interrupted by the martensite sub-microstructure, such as a packet boundary, as shown by the red solid line in Figure 11a and the corresponding experimental counterpart in Figure 11b. However, this packet-scale fractography, which is distinguishable in Figure 8a, may have been the cause of its finer size. This numerical simulation was consistent with the findings that the martensite’s sub-microstructrue contained an effective grain size for toughness [20,21].

**Figure 12:**
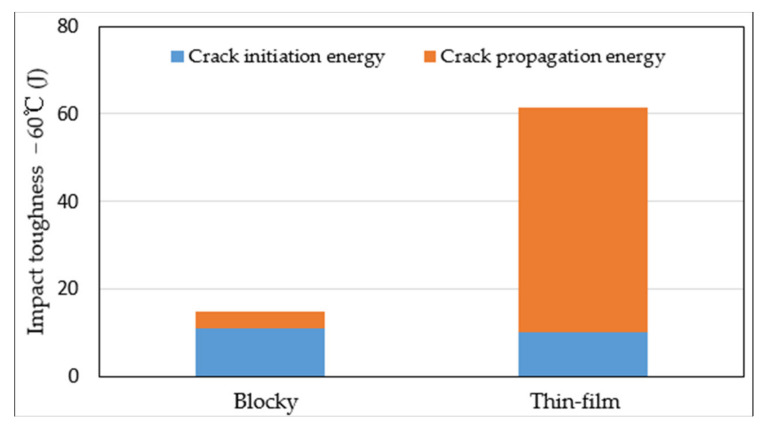
Comparison of fracture energy.

The energy dissipation when the crack encountered blocky MA is shown Figure 12. As compared to that in the thin-film austenite case, the crack-initiation energy in the blocky case was almost identical, whereas its propagation energy was significantly smaller. This investigation was consistent with the crack-propagation resistance, other than initiation energy, enhancing the toughness [17,23]. Our experimental study also showed that the initiation energies that dissipated in the soft and hard microstructure features were almost equal [24]. It is therefore reasonable to infer that the elimination of brittle blocky MA in HAZ was another factor that contributed to the toughness enhancement.

### 4.2. Electro-Thermomechanical Mechanism of RA under EP

The microstructural refinement illustrated in Figure 5 was similar to that in previous findings in which thermal cycles above Ac_1_ were introduced by EPT [10,11]. However, the spotty appearance with a higher austenite fraction shown in Figure 4c was unexpected. Since the concentrated distribution of austenite coincided with the scattered distribution of MA, the spotty morphology was associated with the electrical properties of the MA constituent in contrast with the matrix.

#### 4.2.1. The Local Austenitization

The degradation of Ac_1_ due to a denser dislocation density of MA is a factor responsible for local austenitization. The Ac_1_ is approximately dependent on the free energy change (ΔG), which includes the elastic-strain-free energy and the chemical-free energy. While in a current-carrying system, additional electrical free energy ($\Delta G_{E}$) is supplied in the form of [25]:$\;\Delta G_{E}=\mu_{0}g\xi \left(\sigma_{1},\sigma_{2}\right)j^{2}\Delta V$, where,$\;\mu_{0}$ is the magnetic permeability in vacuum, *g* is a positive geometric factor, and ΔV is the volume of the phase formed during transformation. The coefficient $\xi \left(\sigma_{M},\sigma_{\gamma }\right)$ is a factor that depends on the electrical properties of the phases, and can be defined as [26]: $\xi \left(M,\gamma \right)=\frac{\sigma^{M}-\sigma^{\gamma }}{\sigma^{M}+2\sigma^{\gamma }}$, where $\sigma^{\gamma },\sigma^{M}$ are the electrical conductivity of austenite and martensite in the case of M→γ. According to quantum theory, the electrical resistance $R_{D}$ is created because dislocations render a crystal imperfect, and can be expressed as $R_{D}=\rho_{D}L/A$, where $\rho_{D}$ is the electrical resistivity [27]. The dislocation density of martensite, $\rho_{D}{}^{M}$, is higher than that of austenite, $\rho_{D}{}^{\gamma }$, due to supersaturated lattice of martensite; i.e., $\rho_{D}{}^{M}>\rho_{D}{}^{\gamma }$[14], which gives $\sigma_{M}<\sigma_{\gamma }$, then $\xi \left(M,\gamma \right)<0$. So, $\Delta G_{E}<0$, which indicates that in a current-carrying system, the EPT introduced the electric current free energy, which decreased the barrier of the phase transformation from martensite to austenite, leading to a degradation of Ac_1_. Further, compared to a relatively homogeneous martensite matrix with a dislocation density of $\rho_{D}{}^{M}=$3.5–9.1 × 10^13^ m^−2^ [28], the MA with soft austenite was subjected to a larger crystal deformation, which scattered more electrons, resulting in a higher dislocation density of $\rho_{D}{}^{MA}=$4.6–6.3 × 10^14^ m^−2^ [21], thus:

(1) $$\rho_{D}{}^{MA}>\rho_{D}{}^{M}$$

then, $\sigma^{MA}<\sigma^{M}$, so it can be inferred:

(2) $$\Delta G_{E}{}^{MA}<\Delta G_{E}{}^{M}$$

Equation (2) indicates the MA constituent was more susceptible to austenitization than the martensite matrix with a further decreased Ac_1_.

On the other hand, the EPT-introduced temperature elevation, ΔT, can be expressed as [29]:

(3) $$\Delta T=\frac{Q}{c_{\rho }\cdot d\cdot A\cdot L}$$

where $c_{\rho }$ and  d are the specific heat and mass density; *L* and *A* are the length and area of the sample, respectively; and *Q* is Joule heat, and can be calculated as:

(4) $$Q=j^{2}R_{D}t_{d}$$

Given Equations (3) and (4), the relative temperature elevation between the microfeatures of the MA constituent and the martensite matrix can be expressed as:

(5) $$\Delta T_{MA}>\Delta T_{M}$$

Given the dislocation density value [21,28], it is reasonable to infer that $\Delta T_{MA}=5\Delta T_{M}$. This difference in temperature rise can explain the gradient morphology that the MA constituent experienced during austenitization (spotty morphology shown in Figure 3 and Figure 4) while the martensite matrix underwent recrystallization (nonuniform dimple shown in Figure 8a), and the region adjacent to EP centerline remained heat-untouched due to the lower electrical density (brittle morphology shown in Figure 8b,c). This also explains the distance-dependent microhardness distribution shown in Figure 9a, and the EPT number-dependent toughness shown in Figure 9b. In this regard, the measurement of the K-type thermocouple was not sensitive to temperature at the microscale.

Similar to the evolution of the MA constituent, interfaces or grain boundaries rich in alloy elements in the HAZ microstructure also possess a higher dislocation and lower phase transformation temperature [30], which also were contributors to the higher fraction of austenite along the EP centerline.

**Figure 13:**
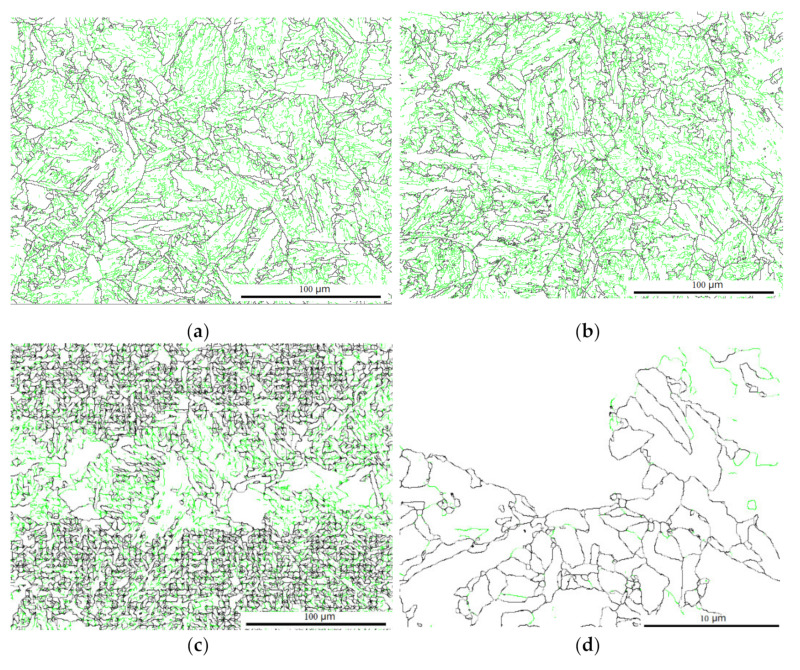
Dislocation density (green line) distribution in the (**a**) as-welded; (**b**) typical; and (**c**,**d**) spotty cases, and its magnification.

In addition to the indirect evidence of metallurgy, phase distribution, and fractography, the theoretical analysis above regarding the preferred Joule heating can also be supported by the heterogeneous evolution of dislocation. Dislocation was measured by EBSD counting small boundaries with angles less than 2.5°, as shown by the green line in Figure 13. The mean dislocation density fraction in the as-welded case exhibited 61.7%. EPT generated a microstructure with relatively low dislocation densities of 56.4% and 42.0% for the typical and spotty cases, respectively. This result quantified that the EPT tended to modify the microstructure with a lower electrical resistivity. This observation was consistent with earlier results [12] in which EP rearranged the microstructure with a lower electrical resistance.

A further low dislocation density with a value of 24.1% was observed in the magnified spotty case, as shown in Figure 13d. The high electrical resistance of brittle factors, such as the MA constituent and boundaries, were favorable for Joule heating, highlighting the capacity of the EP to capture the microstructure heterogeneous in electrical resistivity, in contrast with widely used heat treatment.

#### 4.2.2. The Stability and Appearance of RA

It is well known that reverse austenite is metastable and susceptible to martensite transformation when below $M_{s}$. In fact, EPT provides conditions for RA stability, such as chemical composition and stress states, similar to classical phase transformation [31]. However, this stability is dissimilarly concentrated. The high dislocation features, such as the MA and high alloyed interface or boundary, are rich in alloy elements such as C and Mn [32].

On the one hand, the concentrated temperature elevation at high dislocation density sites generated further atomic enrichment. According to the Boltzmann distribution law, temperature-dependent atomic diffusion $D_{1}$ can be expressed as:

(6) $$D_{1}=D_{0}\exp\left(-\frac{Q_{e}}{kT}\right)$$

where$\;D_{0}$ is the diffusion efficiency, *k* is a constant, $Q_{e}$ is the activation energy, and *T* is the temperature.

Assuming only a 100 °C temperature gradient between the high dislocation features and matrix, the diffusion *D*_1_ in the high-temperature region would be almost three times that in the matrix.

On the other hand, it was reported that a current density larger than ~0.1 A/mm^2^ can initiate atomic diffusion [33]. According to the Nernst–Einstein equation, the atomic diffusion due to an electric field, *D*_2_, can be expressed as:

(7) $$D_{2}=\frac{DNV}{T}$$

where *N* is the atomic density, *D* is the diffusion coefficient, and V is the voltage.

Equation (7) shows that the atomic diffusion *D*_2_ was voltage-dependent. The high dislocation features were subject to higher voltage, and therefore yielded higher atomic diffusion. Equations (6) and (7) indicate that rather than general thermal-induced isotropic atomic diffusion, the high dislocation features are atomic-migrating orientated in a current-carrying system. It is well known that carbon possesses better mobility than Fe due to its smaller atomic size. The supersaturated carbon in high dislocation features orient toward internal austenite, leading to carbon enrichment in reverse austenite. According to the theoretical thermodynamic stability of retained austenite [34], $M_{s}=539-423C-30.4\mathrm{Mn}-17.7\mathrm{Ni}-12.1\mathrm{Cr}-7.5\mathrm{Mo}$. The $M_{s}$ may decrease to below room temperature with a carbon enrichment of 0.8 wt %. Moreover, the larger the size of the high dislocation features, the larger the ΔT and sufficient time for carbon diffusion, and the higher the carbon content of the RA. Additionally, the refined austenite grain size shown in Figure 5a was also a contributor to its stabilization, with the degradation of $M_{s}$ due to an increased phase barrier [35].

Similar to classical phase transformation under fast cooling, compressive stress is generated in RA due to volume expansion because FCC is more closely packed than either BCC or BCT iron, which helps to stabilize RA [36]. Dissimilarly, this expansion is localized, which is expected to introduce higher compressive stress and therefore higher RA mechanical stability.

Additionally, in a current-carrying system, heating speed at scales of above 1000 K/s generates instantaneous maximum compressive stress, given by: $\sigma_{max}=\alpha E\Delta T_{max}$, where E is the elastic modulus, α is the expansion coefficient, and $\Delta T_{max}$ is the maximum temperature elevation. The maximum theoretical compressive stress is 870 MPa. A comparable value of 790 MPa was found in an EP crack-healing investigation [12]. This high compressive stress contributes to RA rearrangement in the appearance of a thin film [37].

## 5. Conclusions

EP was utilized to improve the toughness of an HSS HAZ without polluting the adjacent microstructure. The mechanism behind the toughening was correlated, and the main conclusions were as follows:

1. The MA in HAZ characterized by a rich chemical composition and high dislocation density, and therefore electrical resistivity, enabled concentrated Joule heating and lowered Ac_1_, facilitating a preferred austenitization compared to the matrix recrystallization below Ac_1_ during the EPT, which eliminated the MA constituent.
2. When subjected to EP-introduced undercooling, the reverse austenite evolved to a refined microstructure with an average grain size of 7.07 μm to 4.26 μm, accompanied by the elimination of MA, which improved the toughness of HAZ from 34.1 J to 51.8 J.
3. The effect of EPT toughening faded away with increasing distance from the EP centerline, indicating an effective method for toughening the microstructure within a limited region with a width less than 2 mm.

## Figures and Tables

**Table 1:** Chemical composition of FH690 offshore steel (wt %, balance Fe).

C	Si	Mn	S	P	Ni	Cu + Cr + Mo	Nb + V + Ti
0.08	0.15	1.58	0.002	0.003	0.8	2.3	0.135

## Data Availability

Data sharing not applicable.
